# Engaging Stakeholders With Professional or Lived Experience to Improve Firearm Violence Lexicon Development

**DOI:** 10.2196/68105

**Published:** 2025-04-21

**Authors:** Nicole Cook, Frances M Biel, Kerry Ann Bet, Marion R Sills, Ali Al Bataineh, Pedro Rivera, Anna R Templeton, Natalie Cartwright

**Affiliations:** 1 OCHIN, PO Box 5426, Portland, OR, 97228, United States, 1 9546126511; 2 Norwich University, Northfield, VT, United States

**Keywords:** firearm violence, firearm injury, stakeholder engagement, natural language processing, electronic health record

## Abstract

Framing the public health burden of firearm violence should include people with secondary exposure to firearm violence beyond acute bodily injury, yet such data are limited. Electronic health record clinical notes, when leveraged through natural language processing (NLP), are a potential source of data on firearm exposure. As part of NLP lexicon development, diverse stakeholders were engaged to identify keywords, and our findings demonstrated that engaging diverse stakeholders adds valuable input to NLP development.

## Introduction

Exposure to firearm violence includes witnessing a shooting, being threatened by a firearm, losing a loved one to gun violence, and sustaining injuries from firearms [1]. Such exposure is associated with adverse physical and behavioral impacts. To better understand health sequelae following firearm violence exposure, some researchers recommend including these exposures in ongoing surveillance and research [2,3]. Although structured data (eg, diagnostic codes) for exposure to firearm violence are largely unavailable, unstructured electronic health record (EHR) data represent a potential source of information collected in the clinical care process [4]. To explore the applicability of such data in surveillance and research, we developed a natural language processing (NLP) pipeline for using unstructured EHR fields, including clinical notes, to identify those with exposure to firearm violence and subsequently understand more about the health impacts of such exposure. A crucial part of formative work on NLP lexicon development is effectively engaging a broad representation of stakeholders to contribute lexicon terms that might not otherwise be known to study teams [5]. In this research letter, we describe the process and outcomes of engaging stakeholders in NLP lexicon development.

## Methods

### Study Design

We assembled a stakeholder advisory committee (SAC) comprised of patients, advocates, clinicians, researchers, and others with lived and/or professional experience that includes exposure to firearm violence. The SAC structure was designed to include community advocates, patients with lived experience, practicing clinicians, clinical researchers, data scientists, and clinical informaticists. SAC members were recruited via prior collaboration with a study team member, word of mouth, and presentations to patients and clinicians at web-based workgroup meetings. The final SAC composition included 12 members—5 community advocates and/or patients with lived experience, 1 physician, 2 clinician researchers, 2 clinical informaticists, and 2 data scientists.

In April 2024, SAC members reviewed the lexicon of identified keywords that was developed by the study team [4] and informed by MacPhaul et al’s [6] NLP study, which investigated firearm injury intent. Afterward, SAC members suggested additional terms from their lived or professional experience that may indicate exposure to firearm violence. The review of terms and suggestion of new terms were done via an asynchronous, interactive SAC meeting.

New terms identified by the SAC were searched across EHR clinical notes from 7,103,301 patients who were receiving primary or behavioral health care at community-based health centers in the OCHIN multistate network [7]. A maximum of 30 random notes per keyword were reviewed by a study team member to determine whether a note indicated exposure to firearm violence from a powder mechanism [8]. Descriptive statistics were used to summarize results in a table for review, discussion, and adjudication by the study team and SAC.

### Ethical Considerations

The Norwich University institutional review board deemed this study exempt. Stakeholders agreed to guide this study and received compensation for participating in monthly meetings; they were not research participants. Stakeholders completed a professional services agreement, which outlined roles and responsibilities, and a nondisclosure agreement prior to joining the SAC. The EHR study data used by the study team for NLP development were a limited dataset. SAC members were not provided access to the limited dataset.

## Results

**Figure 1.**
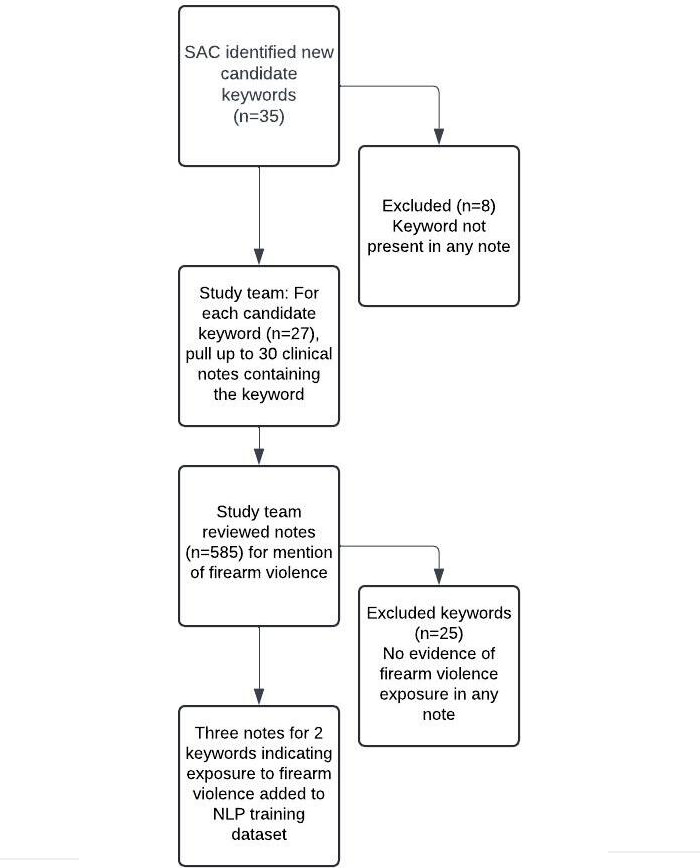
Diagram of SAC keyword term review for firearm violence exposure. NLP: natural language processing; SAC: stakeholder advisory committee.

The initial lexicon of identified keywords provided by the study team to the SAC included 19 terms, of which 13 were used in pilot work and 6 were additionally identified by the study team [4]. SAC members identified 35 additional keywords that were not included in the first iteration of the lexicon. Of the 35 terms, 27 had at least one mention in at least one clinical note. Of the 585 clinical notes reviewed, 5 contained 4 SAC-identified terms*—bbs*, *buckshot*, *firing*, and *metal pole*—and possibly indicated exposure to firearm violence. The study team met to perform the final adjudication for including or excluding each term. Two terms (*buckshot* and *firing*) were determined to have sufficient contextual information to indicate true exposure (Table 1). Notes that included these terms were added to the NLP testing and training dataset (Figure 1).

**Table 1.** Manual review of electronic health record (EHR) clinical notes (N=585) containing stakeholder advisory committee (SAC)–identified firearm lexicon terms from 300 ambulatory health care clinics.

SAC-identified term	Notes in EHR with keyword, n	Clinical notes reviewed^a^^,^^b^, n	Notes with possible indication of exposure to firearm violence, n	Text portion reviewed by study team to determine exposure	Final determination that reviewed clinical notes indicated firearm violence exposure
*buckshot*	1	1	1	“My dad had just been shot in the back, he had buckshot in him.”	Yes
*firing*	13	13	2	“He reports there was a firing of fire arms at his apartment complex this last weekend.” “A few days later, the same brother was apparently responsible for firing a gun into the house; the bullet traversed two rooms and came to rest very close to the patient’s son.”	Yes
*metal pole*	2	2	1	“Pt believes she is being tracked to be murdered, pt sees others are potential threats, as people who are trying to kill her- …reports pt threatening another wielding a metal pole”	No
*bbs*	30	30	1	“Cl said he had a gun and he was not going to jail. (He had a BB gun, broken, threw it in the woods)”	No

a All notes for terms that had less than 30 notes were reviewed. For terms with >30 notes, a random sample of notes was reviewed.

b Additional stakeholder advisory committee–identified terms with clinical notes that did not indicate firearm violence exposure include *Banger*, *draco*, *semiautomatic*, *gat*, *toaster*, *drill*, *stick*, *strap*, *trigger*, *heat*, *heater*, *iron*, *metal*, *nina*, *nine*, *piece*, *pole*, *rod*, *burner*, *cap*, *carrying*, *69*, and *ammunition*.

## Discussion

Effectively engaging varied stakeholders in NLP lexicon development led to the identification of 2 additional terms (*buckshot* and *firing*) that were previously not considered by the study team. Novel clinical notes with these keywords were input into the training and testing dataset to enhance NLP model performance. Although most words suggested by the SAC did not indicate true exposure, the exercise demonstrated that periodic engagement of different stakeholder advisors in artificial intelligence and machine learning research is an important strategy to reduce data bias [9,10].

## Abbreviations

EHR: electronic health record
NLP: natural language processing
SAC: stakeholder advisory committee
